# Multifunctional antibacterial bioactive nanoglass hydrogel for normal and *MRSA* infected wound repair

**DOI:** 10.1186/s12951-023-01929-9

**Published:** 2023-05-21

**Authors:** Long Zhang, Wen Niu, Yuyao Lin, Junping Ma, Tongtong Leng, Wei Cheng, Yidan Wang, Min Wang, Jingya Ning, Shuanying Yang, Bo Lei

**Affiliations:** 1 Department of Respiratory and Critical Care Medicine, The Second Affiliated Hospital of Xiʹan Jiaotong University, Xiʹan, 710004 China; 2 Frontier Institute of Science and Technology, Xiʹan Jiaotong University, Xiʹan, 710054 China; 3 Department of Plastic, Aesthetic and Maxillofacial Surgery, The First Affiliated Hospital of Xiʹan Jiaotong University, Xiʹan, 710061 China; 4 Honghui Hospital, Xiʹan Jiaotong University, Xiʹan, 710068 China

**Keywords:** Bioactive materials, Multifunctional hydrogel, Bioactive glass, Anti-inflammatory, Wound repair

## Abstract

Large-scale skin damage brings potential risk to patients, such as imbalance of skin homeostasis, inflammation, fluid loss and bacterial infection. Moreover, multidrug resistant bacteria (MDRB) infection is still a great challenge for skin damage repair. Herein, we developed an injectable self-healing bioactive nanoglass hydrogel (FABA) with robust antibacterial and anti-inflammatory ability for normal and *Methicillin-resistant Staphylococcus aureus* (*MRSA*) infected skin wound repair. FABA hydrogel was fabricated facilely by the self-crosslinking of F127-CHO (FA) and alendronate sodium (AL)-decorated Si-Ca-Cu nanoglass (BA). FABA hydrogel could significantly inhibit the growth of *Staphylococcus aureus*, *Escherichia coli* and *MRSA *in vitro, while showing good cytocompatibility and hemocompatibility. In addition, FABA hydrogel could inhibit the expression of proinflammatory factor TNF-α and enhance the expression of anti-inflammatory factor IL-4/ IL-10. Based on its versatility, FABA hydrogel could complete wound closure efficiently (75% at day 3 for normal wound, 70% at day 3 for *MRSA* wound), which was almost 3 times higher than control wound, which was related with the decrease of inflammatory factor in early wound. This work suggested that FABA hydrogel could be a promising dressing for acute and *MRSA*-infected wound repair.

**Graphical Abstract:**

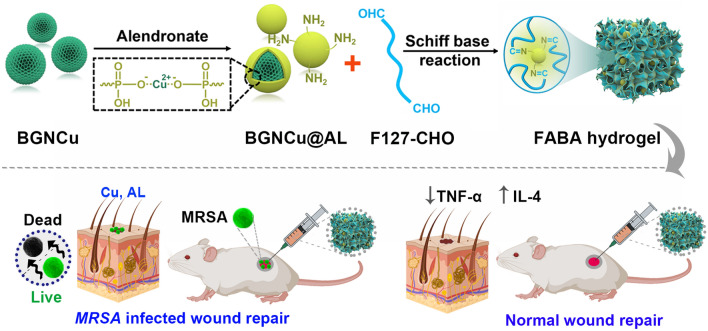

**Supplementary Information:**

The online version contains supplementary material available at 10.1186/s12951-023-01929-9.

## Introduction

Acute and chronic wounds have brought the important threats and economic burdens to human health. Especially, the multidrug resistant bacteria (MDRB) infection wound repair is still a great challenge in the world. Wound healing is a relatively complex process that includes inflammation, cell proliferation and remodeling of the epidermis and subcutaneous tissues [1–4]. Inflammatory phase is an important process in the process of wound healing, but excessive or sustained inflammatory reaction is not conducive to wound healing [5]. In the inflammatory phase, macrophages participate in the regulation of skin wound healing by secreting a variety of cytokines [6]. And numbers of studies proved that regulating the inflammatory response of damaged skin is essential for skin repair [6, 7]. In addition to inflammation, potential wound infection caused by bacteria is another important factor affecting the skin repair process. And large numbers of people die from wound infection all over the world every year [8]. Broad spectrum antibiotics can effectively inhibit the growth of bacteria and minimize wound infection. However, due to the abuse of broad-spectrum antibiotics and the increase of bacterial resistance, antibiotics are not always recommended [9–12]. Therefore, the development of a multifunctional biomaterials dressing that can inhibit drug-resistant bacteria infection and promote wound repair is important and necessary.

Bioactive glass (BG) is an inorganic amorphous biomaterial that composed of CaO (calcium oxide), SiO_2_ (silica) and P_2_O_5_ (phosphorus pentoxide) [13, 14]. In the fields of dental medical devices and bone repair, bioactive glass materials have been approved by the Food and Drug Administration (FDA), indicating that BG has good safety and clinical effects [15]. Compared with traditional BG, bioactive glass nanoparticles (BGN) have been widely used in the field of biomedicine based on the advantages of controllable nanostructure, simple synthesis process, biodegradability, and low cost. And our team has developed multifunctional BGNs for drug delivery, tissue engineering, tumor therapy and wound repair [14, 16–19]. In particular, BGN can be endowed with good antibacterial and anti-inflammatory properties in skin repair through different modifications [14, 16, 20]. As a commercial member of the PEO-PPO-PEO triblock copolymer family, Pluronic^®^F127 (F127) [21] is widely used in burn treatment [22] and anti-inflammatory sustained release carrier [23]. In addition, due to the simple preparation process of the drug-loaded F127 hydrogel, it can effectively avoid drug inactivation or denaturation caused by complex gelation reactions [21, 24]. The aldehyde functionalized F127 (F127-CHO) was selected as chose as the cross linker due to its temperature sensitivity, unique self-assemble into micelles and ease of further modification [25, 26]. Biomedical hydrogel dressing as a promising scaffold has shown good application in the field of wound repair. Hydrogels show a typical three-dimensional cross-linked network, and biodegradable and injectable hydrogels loaded with drugs are widely used in the field of wound repair [27, 28]. In addition, compared with the pre-formed counterparts, injectable hydrogels can match the shape of the injection chamber, which is conducive to tissue regeneration, and can be universal in any non-standard geometry [29].

**Scheme 1:**
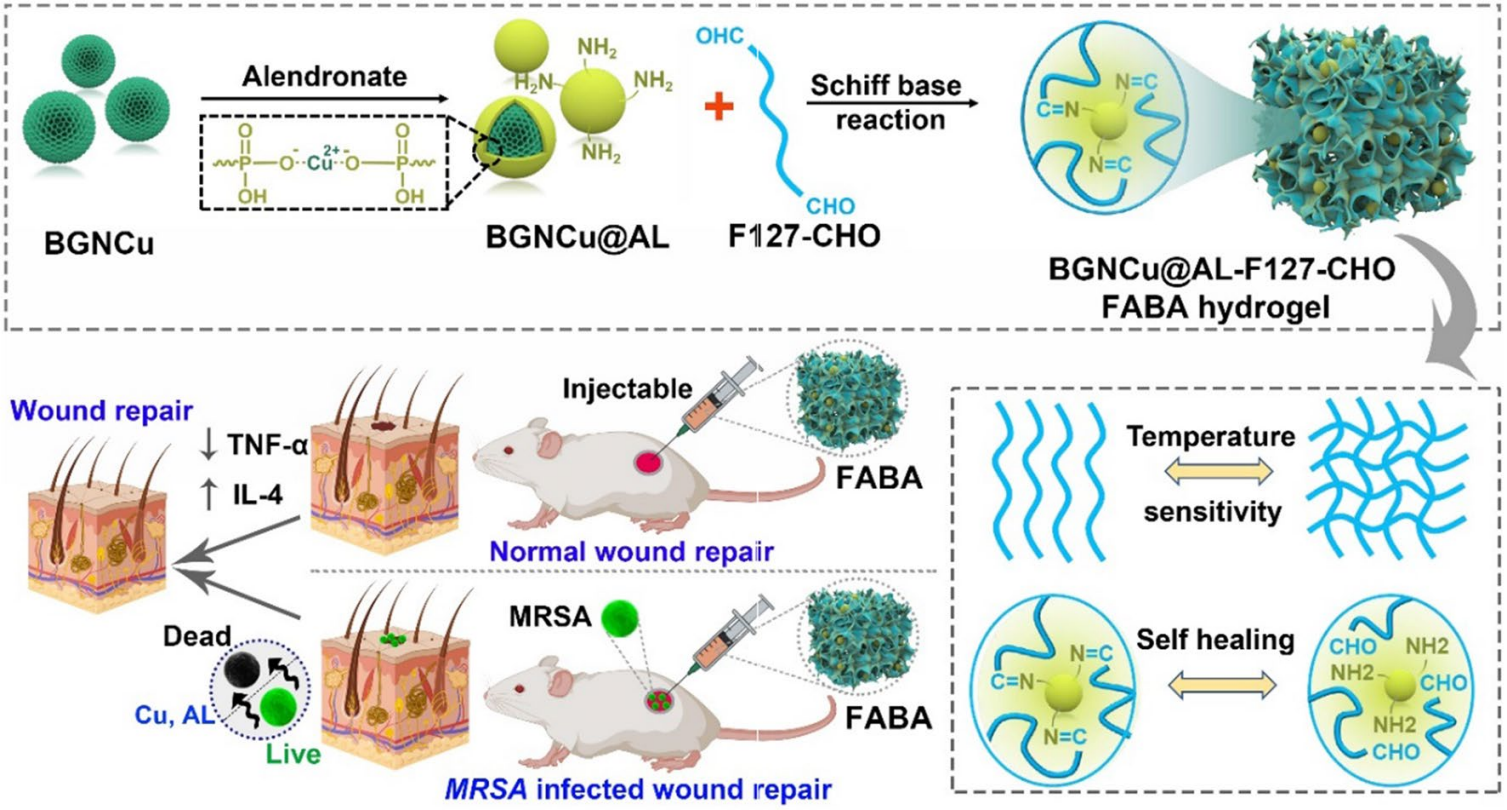
Schematically describe the synthesis of FABA hydrogel and its application in wound healing. This functional FABA hydrogel was fabricated by F127-CHO, BGNCu and alendronate sodium (AL). Leveraging the anti-inflammatory and antibacterial properties of BGNCu and alendronate, this FABA hydrogel showed good performance both in normal wound healing and bacterial infection wound healing

Here, we developed a multi-functional bioactive nanocomposite hydrogel with antibacterial ability and self-repair for normal skin repair and bacterial infection wound healing (Scheme 1). This functional injectable FABA hydrogel was fabricated by F127-CHO and alendronate (AL) sodium decorated BGNCu (BGNCu@AL). In this strategy, the use of BGNCu (amount of copper was doped into BGN) was to take advantage of its antibacterial [30–32] and anti-inflammatory [32–35] properties. By further introducing alendronate sodium with anti-inflammatory [36, 37] and antibacterial [38] properties to increase the performance of the hydrogel. The use of F127-CHO can form a gel network and have a temperature responsive sol-gel conversion ability [39]. The sol-gel behavior, thermosensitivity, injectable, self-healing, antibacterial, cytocompatibility, blood compatibility and *MRSA*-infected wound repair ability of FABA hydrogel were evaluated in detail.

## Results

### **Structure and physicochemical properties of FABA hydrogel**

**Fig. 1:**
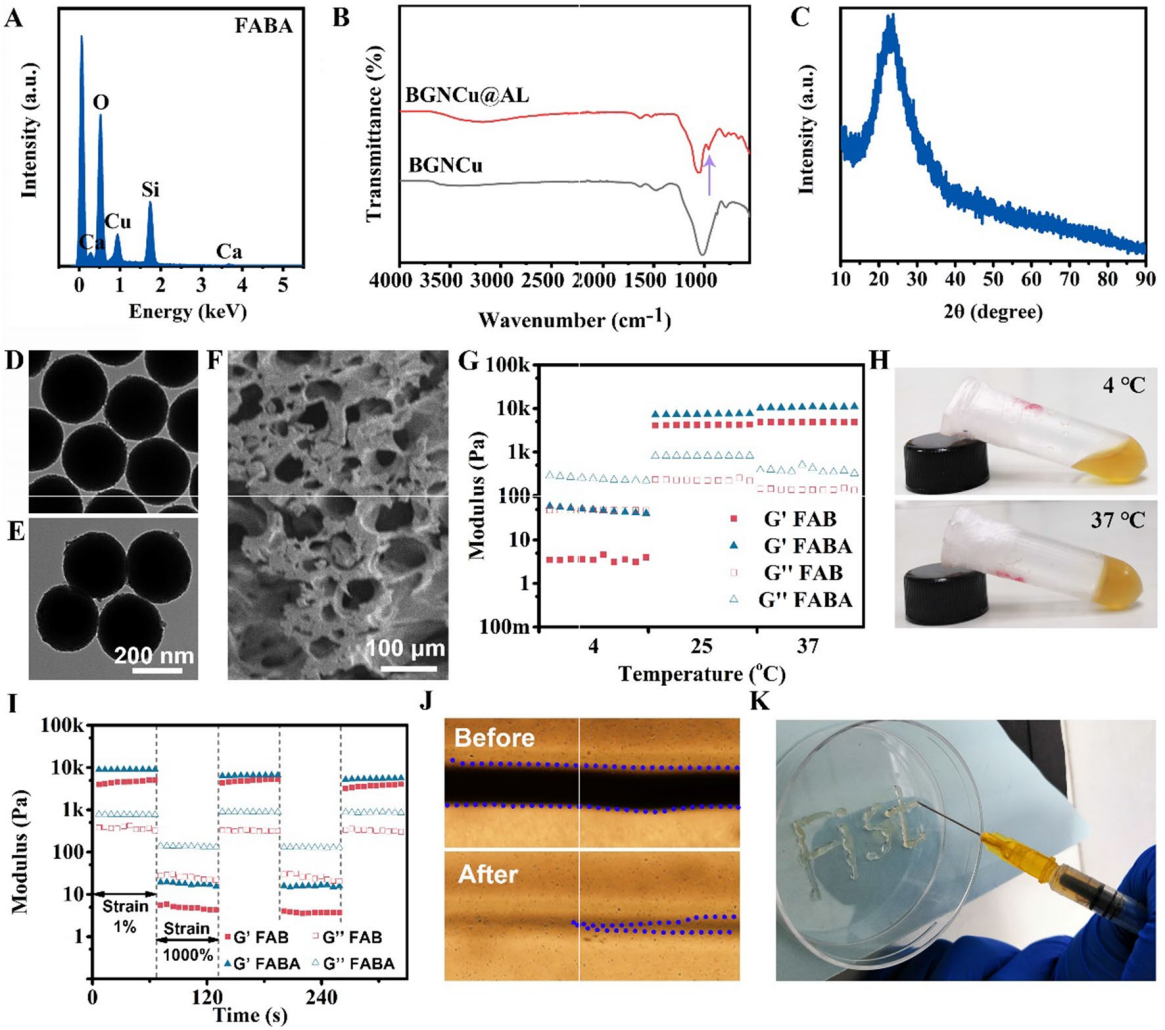
Characterization of BGNCu@AL and FABA hydrogel. **A** EDS spectrum of FABA hydrogel. **B** FT-IR spectrum of BGNCu and BGNCu@AL. **C** XRD of BGNCu@AL. TEM image of BGNCu **D** and BGNCu@AL (**E**). **F** The SEM image of FABA hydrogel. **G** The Gʹ and Gʹʹ at 4, 25 and 37 °C. **H** Thermosensitivity of FABA hydrogel. **I** G’ and G’’ of FAB and FABA hydrogel when the step strain switched from 1–1000% at 37 °C. Self-healing properties (**J**) and injectable properties (**K**) of FABA hydrogel

Figure 1 showed the physicochemical characterization of BGNCu, BGNCu@AL and F127-CHO-BGNCu@AL (FABA hydrogel). The characteristic peaks of Si, Ca, Cu and O/N can be measured in the EDS result (Fig. 1A), indicating that BGNCu@AL which composed of several elements such as Cu, Si, O, N, and Ca exists in FABA hydrogel. And the SEM mapping of BGNCu and BGNCu@AL indicating that BGNCu@AL contained Cu and P (Additional file 1: Figure S1). Previous studies have shown that alendronate can be detected by infrared with P-O bending vibrations of the AL structure at ~ 960 cm^− 1^ [40, 41]. Our FT-IR spectra results of the BGNCu and BGNCu@AL in Fig. 1B indicated that alendronate sodium was coated on BGNCu nanoparticles. As shown in Fig. 1C, the broad peak at 23° corresponded to the amorphous silica, suggesting that Cu doping and AL grafting did not affect the structure of BGN. The particle size of BGNCu (245.01 ± 14.10 nm) and BGNCu@AL (254.67 ± 8.69 nm) were measured by SEM (Fig. 1D, E). SEM result showed that FABA hydrogel has porous structure (Fig. 1F). The FTIR spectra results of FA, FAB and FABA hydrogels showed that the disappearance of the peak at 1735 cm^− 1^ in FABA hydrogel indicating the successful reaction between -CHO in F127-CHO and -NH_2_ in AL (Additional file 1: Figure S2).

In addition, the rheological properties of FABA hydrogel under different conditions were tested (Fig. 1G and I). As the temperature increased from 4°C to 37°C, both FAB and FABA hydrogels G’ increased, indicating that the hydrogels went from sol to gel state. And the G’ values of FAB and FABA did not show significant differences, indicating that the addition of AL did not destroy the temperature sensitivity of the hydrogels (Fig. 1G). Subsequently, a continuous high-low oscillation strain was used to evaluate the self-healing behavior of the FABA hydrogel (Fig. 1I**)**. The G′ dramatically decreased and was lower than the G″ value at the high oscillation strain (1000%), which suggested that the hydrogel network had suffered serious damage. Once the strain was switched to 1%, the recovery of the G′ and corresponding G″ values indicated that the crosslink networks of the hydrogel recovered effectively. Even after two cycles, there was no obvious decrease of the modulus at a strain of 1%, suggesting that the FABA hydrogel has excellent self-healing ability. Since the body temperature in general tends to be slightly below 37 °C, we examined the rheological properties of FABA hydrogels at slightly below 37 °C (at 35 °C, Additional file 1: Figure S3). The results showed no significant change in the storage modulus of FABA hydrogels at 35 °C compared to 37 °C.

FABA hydrogel was placed in a transparent plate, and the self-healing behavior of the hydrogel was observed by microscopy. With the passage of time, the crack of the hydrogel became smaller and disappeared completely after 30 min, indicating that the FABA hydrogel has good self-healing ability (Fig. 1J). In addition, FABA hydrogel is temperature-sensitive, with 4 °C being liquid glue and 37 °C being solid glue (Fig. 1H). And this reversible conversion between solid and liquid is related to temperature. FABA hydrogel can be injected through the syringe, showing good injectable property (Fig. 1K). The injectable properties make the FABA hydrogel perfectly match any shape of wound, which makes its application more convenient. In addition, we also measured the degradation rate of hydrogel FABA in vitro and in vivo. The results showed that FABA was able to degrade completely after 960 min of treatment at pH = 7.4 in vitro (Additional file 1: Figure S4). The FABA hydrogel could be completely degraded 12 h after subcutaneous implantation. And a 50% degradation rate was showed at 3 h (Additional file 1: Figure S5).

### Biocompatibility, anti-inflammatory and antibacterial of FABA hydrogel

**Fig. 2:**
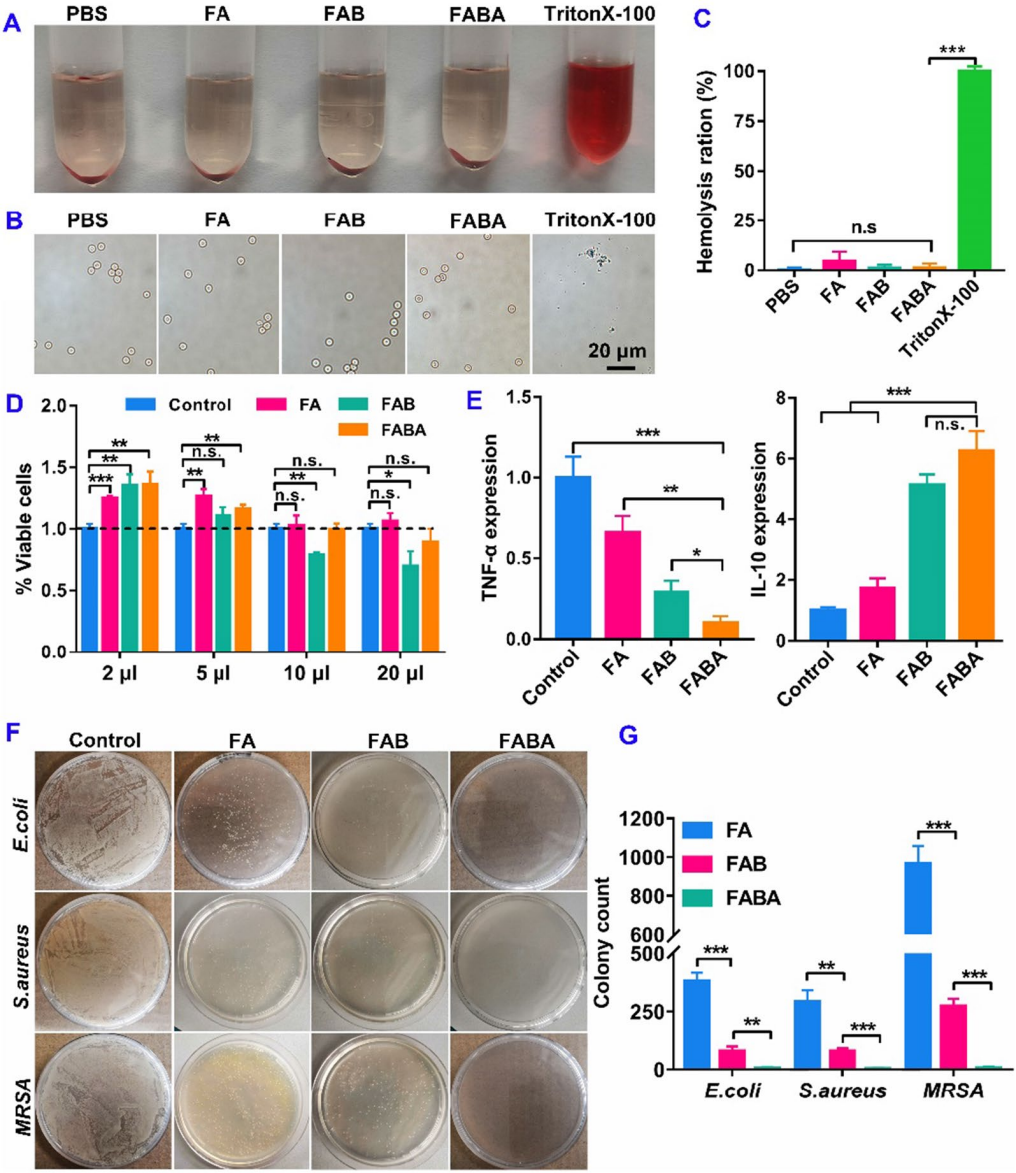
Biocompatibility, anti-inflammatory and antibacterial of FABA hydrogel. **A**, **B** Image of hemolysis of mice red blood cells after hydrogel or tritonX treatment for 1 h. **C** Statistics of erythrocyte hemolysis rate (n = 3). **D**. Biocompatibility of FA, FAB and FABA hydrogels on L929 cells (n = 4). **E** Evaluation of anti-inflammatory properties of FA, FAB and FABA hydrogels in macrophages. *TNF-α* and *IL-10* expression were quantified by RT-qPCR (n = 3). **F** The antibacterial activity of FA, FAB and FABA hydrogels on *E.coli, S.aureus* and *MRSA*. **G** The clone numbers of *E.coli, S.aureus* and *MRSA* after FA, FAB and FABA treatment (n = 3). *P < 0.05, **P < 0.01, ***P < 0.001 and n.s. means no significance

To detect the hemolytic property of FABA hydrogel, red blood cells were incubated with hydrogels and TritonX-100 respectively. After centrifugation, there was no hemolysis phenomenon in the hydrogels treated groups (supernatant colorless and transparent), but obvious hemolysis was observed in the control group treated with TritonX-100 (Fig. 2A). The morphological characteristics of the cells were observed under a microscope. Compared with TritonX group, the erythrocytes in the hydrogels treated groups were intact and no cell breakage was observed (Fig. 2B). The hemolysis rate showed that the FABA hydrogel had extremely low hemolysis rate (less than 4%) (Fig. 2C), which is within the safe range of biomaterials [39, 42]. These results proved that FABA hydrogel not only has splendid antibacterial and anti-inflammatory properties, but also excellent cell and hemo-compatibility. Subsequently, L929 cells were used to detect the cytocompatibility (cytotoxicity) of FABA hydrogel. The cell viability was measured after 24 h incubation with 2 µL, 5 µL, 10 µL, 20 µL of hydrogel in 100 µL cell culture medium respectively. The CCK8 results showed that under different volume ratios, both FABA and FA showed good biocompatibility, while FAB over 10 µL had certain cytotoxicity (Fig. 2D).

To further evaluate the anti-inflammatory properties of hydrogels, FA, FAB and FABA hydrogel was added into LPS-stimulated macrophages separately. RT-qPCR results showed that FABA hydrogel could down-regulated the expression of the pro-inflammation gene *TNF-α* while promoting the anti-inflammatory gene *IL-10* expression in macrophages (Fig. 2E). Compared with FA and FAB, FABA hydrogel exhibited the better anti-inflammatory property. Previous studies have shown that bioactive glass (BG) ion products can effectively stimulate the transformation of macrophages to M2 type [33]. And 45S5 bioglass can reduce the production of *IL-6* and *TNF-α* by macrophage at relatively low concentrations [43]. Lin et al. have shown that Cu-BG can significantly inhibit osteochondral tissue inflammatory response in the treatment of osteoarthritis. In addition to the anti-inflammatory effect of BG, Cu can promote the expression of anti-inflammatory gene *IL-10* and down-regulate the expression of pro-inflammatory gene *IL-1β* when inducing hMSC differentiation [32].

Three different kinds of bacteria such as *Escherichia coli* (*E.coli*, Gram negative), *Staphylococcus aureus* (*S.aureus*, Gram positive) and *Methicillin-resistant Staphylococcus aureus* (*MRSA*, multidrug-resistant) were used to detect the antibacterial properties of FABA hydrogel. After 10^5^ bacteria was added to the surface of 300 µL hydrogel in a 48-well cell culture plate and incubated at 37 °C for 3 h. Subsequently, 100 µL of PBS was gently added into the cell culture plate to resuspend the bacteria. 5 µL of bacterial solution was coated on LB solid culture dish without antibiotics. After incubation at 37 °C for 12 h, the number of monoclone colonies in the culture dish was counted. The results showed that FA, FAB and FABA hydrogel showed certain antibacterial activity. By counting the number of bacteria in the culture plate, FABA exhibited better antibacterial properties compared with FA and FAB groups (Fig. 2F–G).

### Normal acute wound repair evaluation

**Fig. 3:**
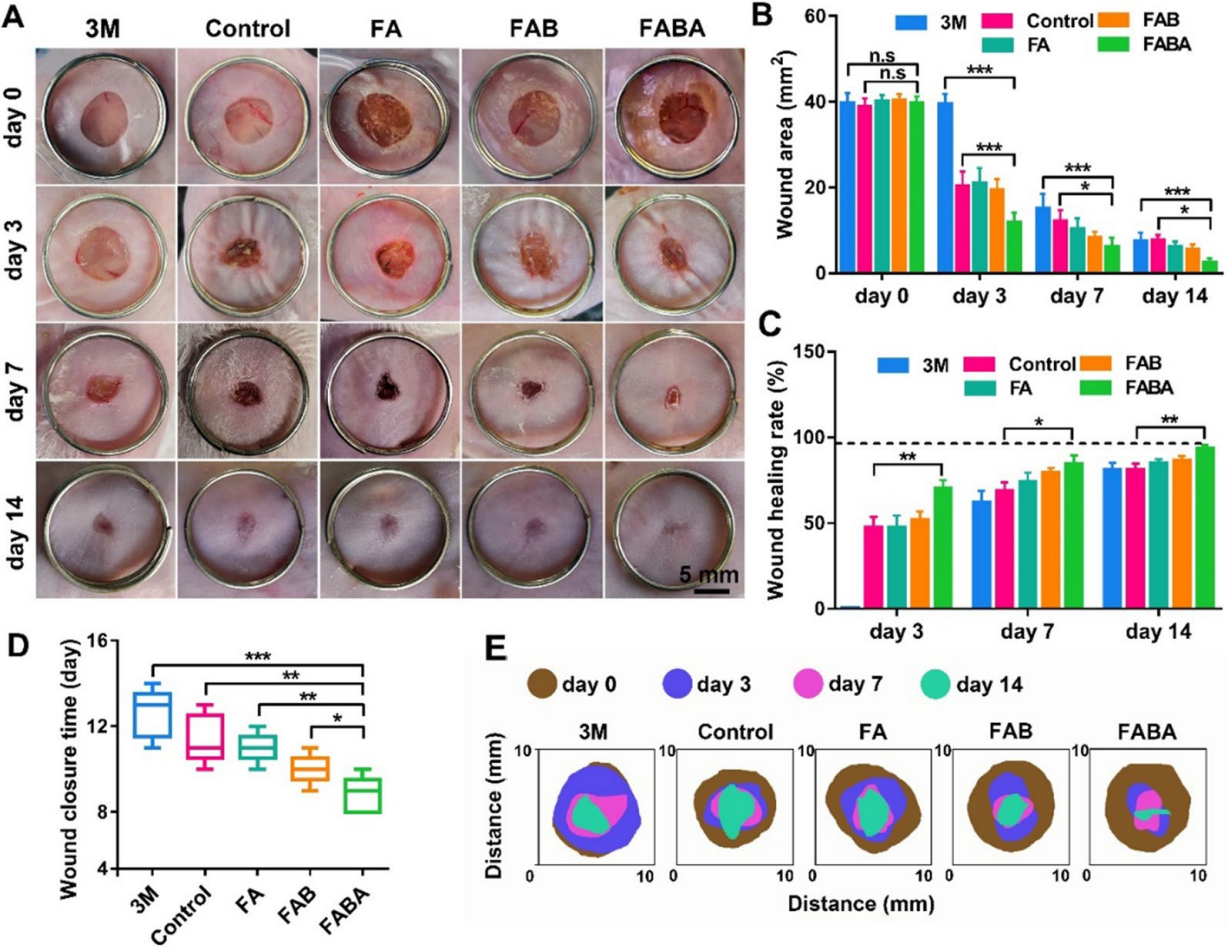
Evaluation of FABA hydrogel on normal skin wound healing in mice. **A** Imaging of wound healing on day 0, 3, 7 and 14 after 3 M, control, FA, FAB and FABA treatment. **B** The wound area on day 0, 3, 7 and 14 after 3 M, control, FA, FAB and FABA treatment. **C** Statistics of skin wound healing rate on day 3, 7 and 14. **D** Statistics of skin wound closure time in different treatment groups. **E** Schematic diagram showed the wound area size on day 0, 3, 7 and 14. n = 6, *P < 0.05, **P < 0.01, ***P < 0.001 and n.s. means no significance

We constructed the acute skin injury model in mice to detect the effect of FABA hydrogel on normal wound healing. Compared with the commercial 3 M Tegaderm film group, the wound area both in the control group, FA, FAB and FABA hydrogels treatment groups decreased significantly on day 3 (Fig. 3A, B). From day 7 to day 14, the wounds area and wound healing rate of mice in each group were rapidly improved until the wound defect was completely closed (Fig. 3A, C). The wound closure time is one of the most important indicators of skin repair, which could greatly reduce the risk of wound infection. By recording the wound closure time, we found that the wound defects in the FABA group could completely closed (skin gap was completely healed, in other words, the subcutaneous tissue and skin gap could no longer be seen after the tissue at the wound site was closed) on day 7 (Fig. 3D). Meanwhile, wound area size on day 0, 3, 7 and 14 was schematically shown in Fig. 3E. Compared with 3 M, control, FA and FAB groups, FABA showed better skin repair efficiency in terms of wound healing rate, healing area analysis and wound closure time.

**Fig. 4:**
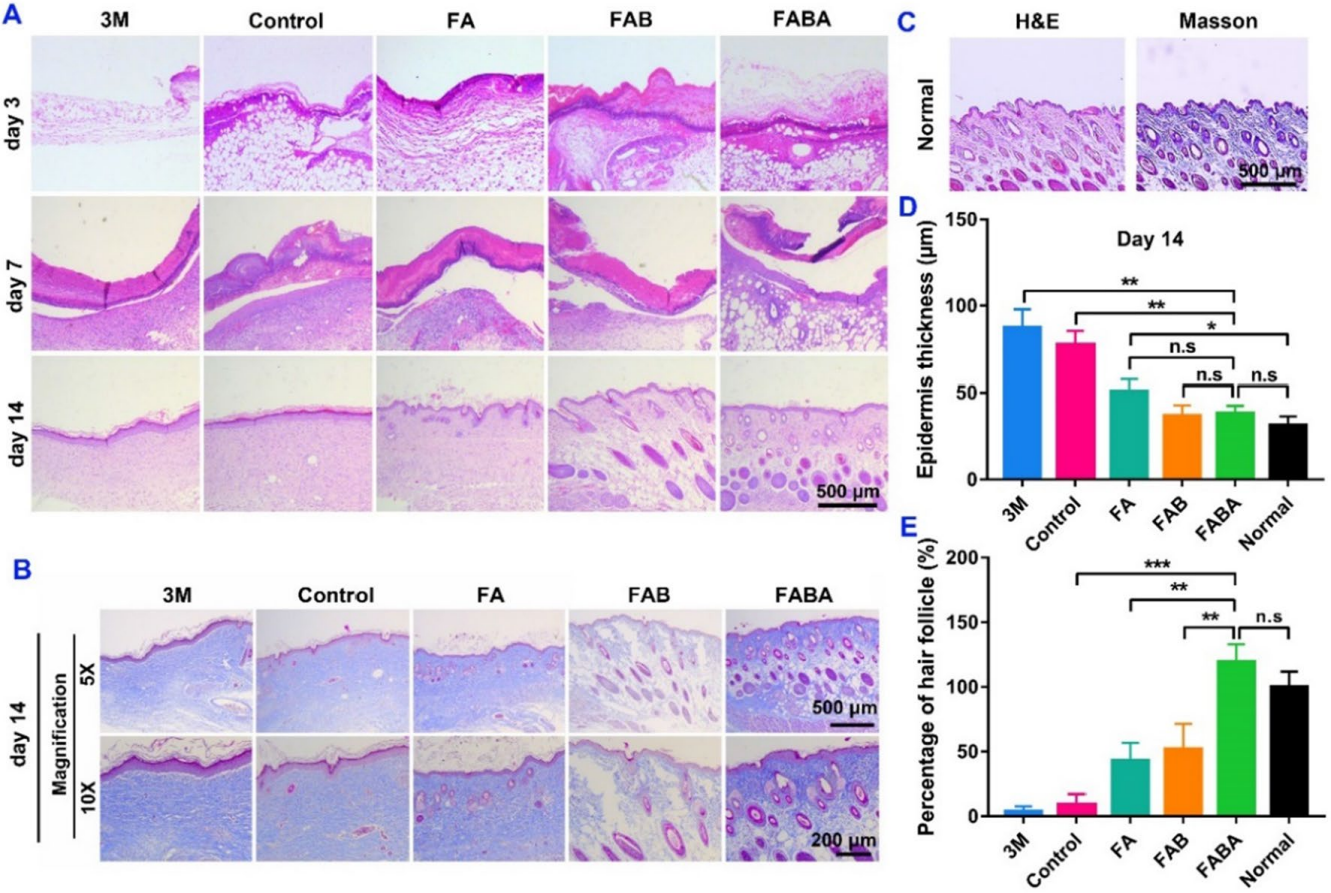
Histological evaluation of the effect of FABA hydrogel on wound healing. **A** The image of wound H&E staining on day 3, 7 and 14. **B** Masson’s trichrome staining of wound on day 14. **C** H&E and Masson’s trichrome staining of normal skin. **D** Statistical results of epidermis thickness on day 14. **E** The percentage of hair follicles (Standardized with normal skin). n = 6, *P < 0.05, **P < 0.01, ***P < 0.001 and n.s. means no significance

To further evaluate the wound healing effect of FABA hydrogel from the histological staining level, wound skin was collected for H&E, immunofluorescence and Masson’s trichrome staining. Compared with the control group, H&E staining showed that the use of FABA hydrogel increased the reconstruction of epidermis and subepithelial tissue (Fig. 4A). On day 7, hair follicles and other skin appendages were observed in the newborn skin of FABA treatment group (Fig. 4A). By counting the skin epidermal thickness in each group on day 14, there was no significant difference between FAB, FABA and the normal tissues (Fig. 4A–D). Both in H&E and Masson’s trichrome staining on day 14, the newborn skin tissue in FABA treated group was more similar to that in normal skin tissue, such as hair follicles, skin appendages and epidermal thickness (Fig. 4A**–**E).

**Fig. 5:**
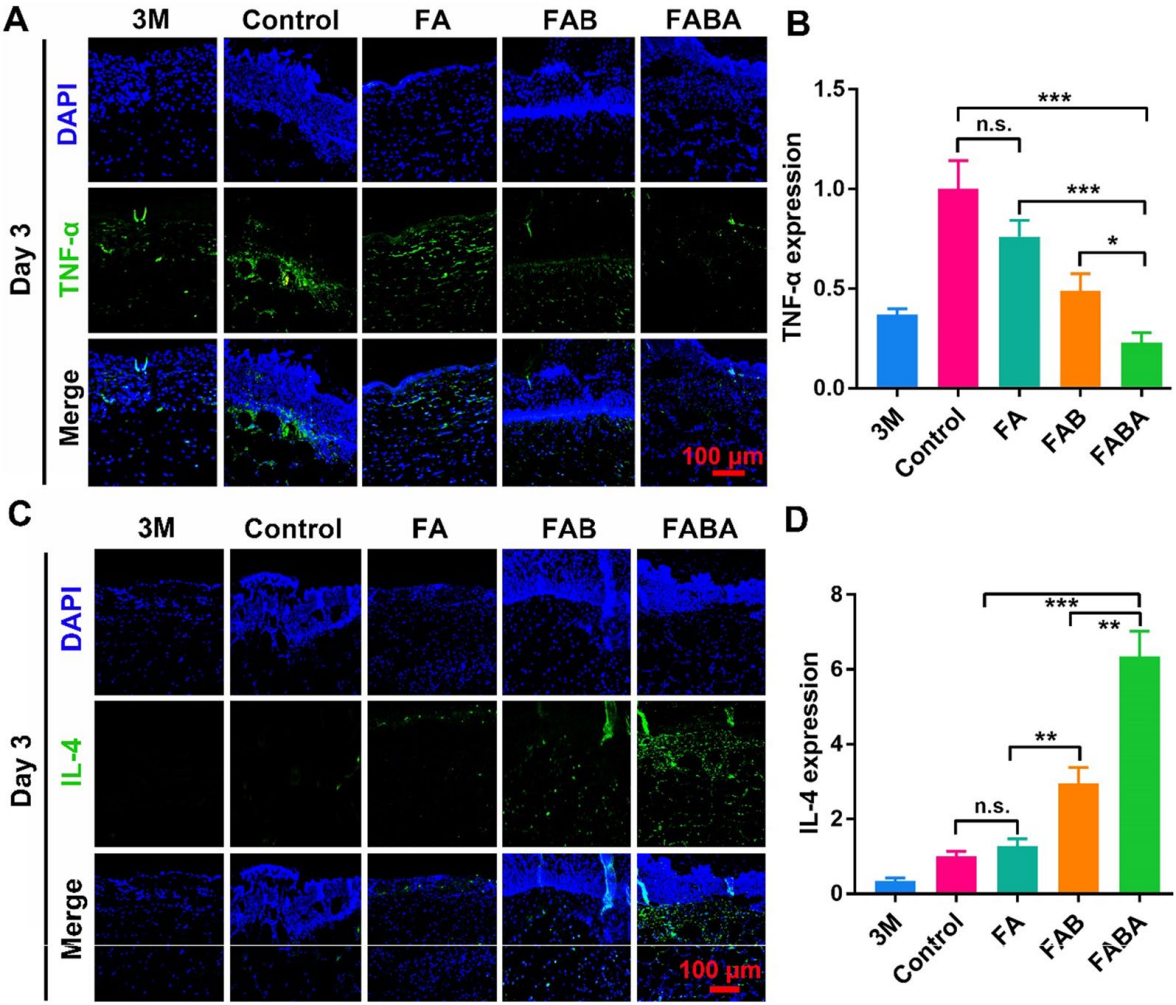
Immunofluorescence staining of TNF-α and IL-4 in wound skin. **A** The expression of TNF-α (green) in wound skin on day 3 was stained (nuclear: blue by DAPI). **B** The intensity of TNF-α immunofluorescence was quantified by ImageJ software. **C** The expression of IL-4 (green) in wound skin on day 3 was stained (nuclear: blue by DAPI). **D** The intensity of IL-4 immunofluorescence was quantified by ImageJ software. n=6, *P < 0.05, **P < 0.01, ***P < 0.001 and n.s. means no significance

Numbers of studies have shown that inflammation was related to skin repair. And skin repair process can be improved by regulating the inflammatory response [7, 44–46]. Here, we evaluated the effect of hydrogel on skin repair by detecting the expression of TNF-α and IL-4. On day 3, the early stage of skin repair, FABA hydrogel could effectively inhibit the expression of pro-inflammatory protein TNF-α (Fig. 5A, B) and promote the expression of anti-inflammatory protein IL-4 **(**Fig. 5C, D). To sum up, in comparison with other control groups, FABA hydrogel has an excellent effect in wound healing and excellent anti-inflammatory effect. It could complete wound closure and skin tissue repair in the shortest time, which dramatically reduced the risk of wound infection.

### *MRSA* infected wound repair

**Fig. 6:**
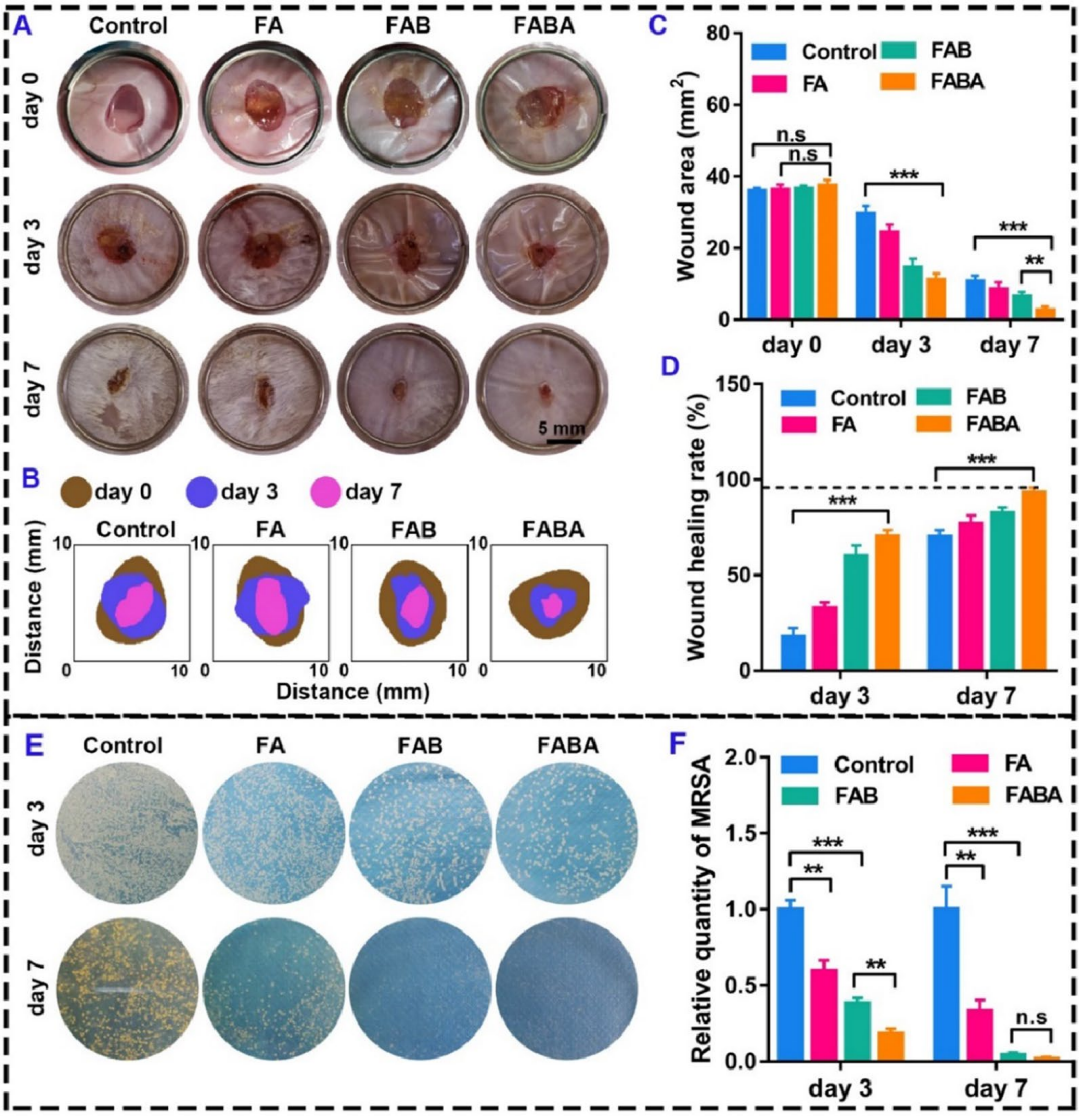
Anti-infective effect of FABA in *MRSA* infection-induced wound healing. **A** Imaging of wound healing on day 0, 3 and 7 after control, FA, FAB and FABA treatment. **B** Schematic diagram showed the wound area size on day 0, 3 and 7. **C** The wound area on day 0, 3 and 7. **D** Statistics of skin wound healing rate on day 3 and 7. **E** The antibacterial activity of FA, FAB and FABA hydrogels on *MRSA* in the infection-induced wound healing at day 3 and 7. **F** The relative clone numbers of *MRSA* on day 3 and 7 (n = 3). **P < 0.01, ***P < 0.001 and n.s. means no significance

Multidrug resistant bacteria (MDRB) infection is one of the major challenges in wound healing [47, 48]. Due to skin damage or deficiency, bacteria can easily break through the natural barrier of the skin and cause wound infection [49]. Therefore, wound healing dressings with antibacterial properties are particularly important in the process of skin repair [50]. Based on the antibacterial properties of FABA hydrogel in vitro, we prepared *MRSA* infection-induced wound healing model mice to evaluate the antibacterial and skin repair capabilities of FABA hydrogel in vivo. In *MRSA* infection-induced wound healing experiment, FABA hydrogel also showed excellent skin repair function. On day 3, the wound area in FABA group was significantly decreased (Fig. 6A–C). Although the wounds of all groups were effectively repaired on day 7, the wound area and wound healing rate of FABA were the best (Fig. 6C, D). After 3 days of *MRSA* treatment, obvious pus exudation was seen in the wounds of the control and FA hydrogel groups, but no obvious pus was seen in the wounds of the FAB and FABA groups (Fig. 6A). And this phenomenon disappeared on day 7. Although *MRSA* grew in all treatment groups on day 3, there was almost no bacterial growth on the LB plate in FABA group on day 7 (Fig. 6E, F). These results showed that FABA hydrogel could control wound bacterial infection in *MRSA* infected wound model. Through H&E staining of infected wound skin on day 3 and day 7, it was found that the skin of mice in the FABA treatment group repaired better than that in the control group (Additional file 1: Figure S6). Main tissue organs (spleen, liver, kidney and lung) were collected for H&E staining. The results showed that FA, FAB and FABA hydrogels showed good tissue safety after 3 d and 7 d of treatment in the *MRSA* infected wound model mice (Additional file 1: Figure S7).

We also used methicillin as the negative control and vancomycin as the positive control to compare the antibacterial properties of FABA hydrogel (Additional file 1: Figure S8). Skin wounds were made on the waist of mice. Then 10 µL of fresh MRSA bacterial (10^8^ CFU/mL) solution was added to the injured skin. Mice were randomly divided into three groups (n = 8). The mice in FABA, methicillin and vancomycin groups were separately treated with FABA hydrogel, methicillin (22.5 µg/mouse) [51] and vancomycin (50 mg/kg) [52]. Mice wound size was counted on day 0, day 3, and day 7, respectively (Additional file 1: Figure S8A-D). The results showed that the wound healing rate was faster in the FABA hydrogel group compared to the methicillin group on day 3 (Additional file 1: Figure S8B-D). However, compared with the vancomycin-treated group, the FABA hydrogel did not show a significant advantage (Additional file 1: Figure S8C-D). On day 3 and day 7, skin samples were collected and vibrated in 1 mL PBS for 30 s. Then 10 µL of bacterial solution was coated on Luria-Bertani (LB) solid culture dish (Additional file 1: Figure S8E). After inubation at 37 °C for 12 h, the number of monoclone colonies in the culture dish was counted (Additional file 1: Figure S8F). On day 3, there was no significant difference in antimicrobial performance between the FABA and vancomycin groups, but on day 7, FABA had a more pronounced antimicrobial advantage (Additional file 1: Figure S8E-F). This may be because the vancomycin-treated group in this study was given only once, whereas clinics usually give multiple consecutive doses. However, both vancomycin and FABA groups showed better antibacterial performance than methicillin group on day3 and day7.

**Fig. 7:**
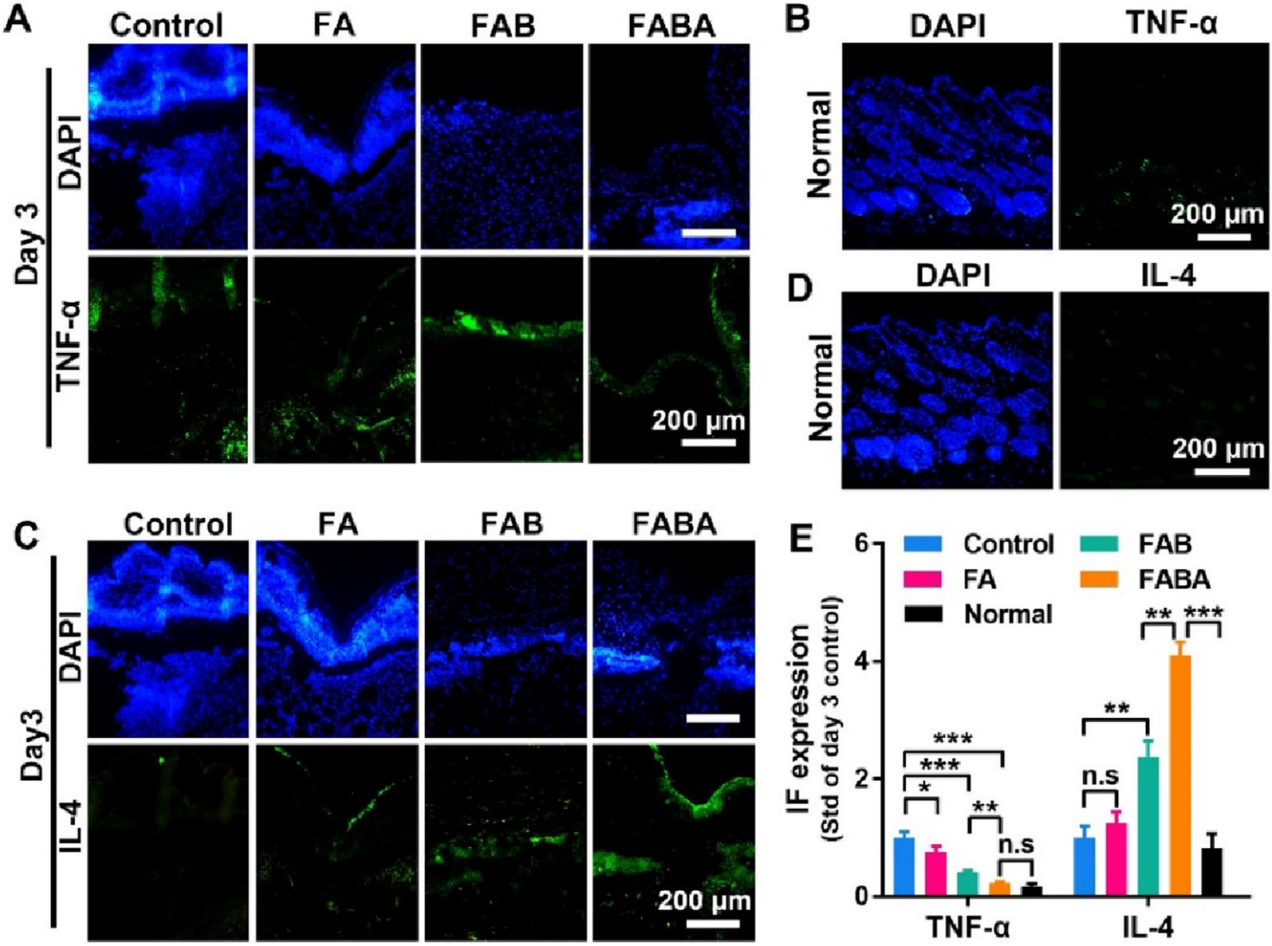
Immunofluorescence staining of TNF-α and IL-4 in *MRSA* infection-induced wound healing. **A** The expression of TNF-α (green) in *MRSA* infection-induced wound skin on day 3 was stained (nuclear: blue by DAPI). **B** TNF-α expression in normal skin tissue. **C** The expression of IL-4 (green) in MRSA infection-induced wound skin on day 3. **D** IL-4 expression in normal skin tissue. **E** The intensity of TNF-α and IL-4 immunofluorescence was quantified by ImageJ software. n=6, *P < 0.05, **P < 0.01, ***P < 0.001*P<0.05, **P<0.01, ***P<0.001 and n.s. means no significance

In addition, we evaluated the effect of FABA hydrogel on *MRSA* infection-induced wound healing model by detecting the expression of inflammatory protein TNF-α and anti-inflammatory protein IL-4. The results showed that FABA hydrogel inhibited TNF-α expression (Fig. 7A–E) and promoted the expression of anti-inflammatory protein IL-4 on day 3 compared with the control group (Fig. 7C–E). Compared with the normal tissue (Fig. 7B), the higher expression of TNF-α expression indicated that the infected wound tissue was still inflamed on day 3. Conversely, the higher expression of IL-4 than that in normal tissue suggested that FABA hydrogel can effectively inhibit inflammation (Fig. 7C–E). These results indicated that FABA can effectively inhibit *MRSA* and promoted skin repair in *MRSA* infected wound healing model.

## Discussion

In this study, a multifunctional bioactive nanocomposite hydrogel with antibacterial and self-healing abilities for normal skin repair and bacterial infection wound healing was facilely fabricated by the self-crosslinking of FA and AL-decorated Si-Ca-Cu nanoglass. Compared with conventional bioactive glass (BG), bioactive glass nanoparticles (BNG) shows the advantages of controllable nanostructure, simple synthesis process, biodegradability, low cost, and can be used for drug delivery, tissue engineering, tumor therapy and wound repair [14, 16–19]. As a commercial member of the PEO-PPO-PEO triblock copolymer family, Pluronic®F127 (F127) [21] is widely used in burn treatment [22] and anti-inflammatory sustained release carrier [23]. F127-CHO was selected as chosen as the crosslinker due to its temperature sensitivity, unique self-assembly into micelles and ease of further modification [25, 26]. By further introducing alendronate sodium with anti-inflammatory [36, 37] and antibacterial [38] properties to increase the performance of the hydrogel. This FABA hydrogel synthesized from F127, BGNCu and AL has sol-gel behavior, heat sensitivity, injectability, self-healing, antibacterial, cytocompatibility, blood compatibility and wound healing ability of *MRSA* infection.

Previous studies have shown that bioactive glass (BG) ion products can effectively stimulate the transformation of macrophages to M2 type [33]. And 45S5 bioglass can reduce the production of *IL-6* and *TNF-α* in macrophage at relatively low concentrations [43]. Lin et al. have shown that Cu-BG can significantly inhibit osteochondral tissue inflammatory response in the treatment of osteoarthritis. In addition to the anti-inflammatory effect of BG, Cu can promote the expression of anti-inflammatory gene *IL-10* and down-regulate the expression of pro-inflammatory gene *IL-1β* when inducing hMSC differentiation [32]. In particular, BGN can be endowed with good antibacterial and anti-inflammatory properties in skin repair through different modifications [14, 16, 20]. In this study, Si-Ca-Cu nanoglass (BGNCu) was used to prepare FAB and FABA hydrogels, and the results showed that the hydrogels doped with BNGCu could effectively inhibit the expression of inflammatory gene TNF-α and promote the expression of anti-inflammatory genes IL-10 and IL-4 (Figs. 2E, 5 and 7). Prolonged inflammation may inhibit wound repair and cause scar formation, which is detrimental to skin repair [5, 7]. And numbers of studies proved that regulating the inflammatory response of damaged skin is essential for skin repair [6, 7]. Based on the dual anti-inflammatory effect of BGNCu [32–35] and AL [36, 37], FABA hydrogel exhibits better anti-inflammatory properties than FAB and has superior results in skin repair processes (Figs. 3, 4 and 6).

In addition to inflammation, potential wound infection caused by bacteria is another important factor affecting the skin repair process. And large numbers of people die from wound infection all over the world every year [8]. Multidrug resistant bacteria (MDRB) infection is one of the major challenges in wound healing [47, 48]. Due to skin damage or deficiency, bacteria can easily break through the natural barrier of the skin and cause wound infection [49]. Therefore, wound healing dressings with antibacterial properties are particularly important in the process of skin repair [50]. In this strategy, the use of BGNCu [30–32] and alendronate sodium [38] were to take advantages of their antibacterial properties. The results showed that the antibacterial performance of FABA hydrogels containing BNGCu and AL was significantly higher than that of FAB hydrogels containing only BGNCu (Figs. 2F, G and 6E, F). And in the *MRSA* infection-induced wound healing experiment, FABA hydrogel effectively inhibited *MRSA*-induced skin wound infection and promoted wound healing (Fig. 6A–D).

Although FABA hydrogel can effectively promote Normal acute wound repair and *MRSA* infected wound repair, there are still some shortcomings in this study, which can be done better in the future. First, although studies have shown that BGNCu and AL can be anti-inflammatory and antibacterial, and our study confirms this, the mechanism of action is not clear. The molecular mechanism of the anti-inflammatory and antibacterial properties of FABA hydrogels needs to be further investigated. Second, although FABA hydrogel is effective in inhibiting skin infection and promoting wound healing in the *MRSA* wound infection model, it is not clear how immune cells such as macrophages, B cells and dendritic cells function in the wound tissue during the in vivo antimicrobial process. These questions deserve a follow-up in-depth study. However, this study demonstrated that FABA hydrogel could promote wound healing with regeneration of skin appendages and wound repair in *MRSA* infections. This study suggests that FABA hydrogel could be a promising dressing for wound repair in acute and bacterial infections.

## Conclusion

In conclusion, the multifunctional injectable self-healing bioactive FABA hydrogel could be facilely fabricated by the self-crosslinking of FA and AL-decorated Si-Ca-Cu nanoglass (BA). FABA hydrogel showed robust antibacterial and anti-inflammatory activity, while keeping the good cytocompatibility and blood compatibility. FABA hydrogel could promote the wound healing with regeneration of skin appendage and *MRSA*-infected wound repair through inhibiting *MRSA* infection, upregulating the expression of anti-inflammatory factor IL-4 and down-regulating the expression of pro-inflammatory factor TNF-α during the wound healing process. This study suggests that FABA hydrogel could be a promising dressing for acute and bacterial infected wound repair.

## Materials and methods

### Synthesis of F127-CHO and FBAB hydrogel

The substrate material F127-CHO of FABA hydrogel was synthesized according to our previous description [25, 53, 54]. More details can be found in the supplementary materials. Then, FABA hydrogel was synthesized by the crosslinking of F127-CHO and BGNCu@AL. Detailed methods were showed in supplementary materials.

### Physicochemical characterization

The element and phase compositions of F127-CHO-BGNCu@AL (FABA) were tested by a scanning electron microscopy (SEM, QUANTA FEG250, Thermo) equipped with the energy-dispersive spectrometer (EDS). A X-ray diffractometer (XRD, D8 ADVANCE, Bruker) with a Ni-filtered Cu Kα irradiation was used to test whether Cu doping and AL grafting will affect the structure of BGN. The morphology of BGNCu@AL was observed using a transmission electron microscope (TEM, JEM-F200, JEOL). The chemical structure of BGNCu@AL was analyzed by a fourier transform infrared spectroscopy (FTIR, Nicolet 6700, Thermo) in transmission mode. The rheological properties of FABA hydrogel under different temperature and oscillatory strain were analyzed by a rheometer (DHR-2, TA). The thermal properties of FABA hydrogels were determined by observing the state of the hydrogels at 4, 25 and 37 °C. The self-healing abilities of FABA hydrogels were measured by the state of the hydrogels at 5 stress change cycles (1-1000% strains).

### Biocompatibility and safety of FABA hydrogel in vitro

Red blood cells were used to detect the hemolytic property of FABA hydrogel. In brief, 200 µL hydrogel and 500 µL diluted red blood cell was co-cultured into the culture plate and 0.1% TritonX-100 were used as control. After incubation at 37 °C for 1 h, the cell suspension was collected to evaluate the hemolysis of red blood cells. Then L929 cells and CCK8 assay Kits (C0005, TargetMol, USA) were used to detect the cytotoxicity of the FABA hydrogel. More details can be found in the supplementary materials.

### Antibacterial and anti-inflammatory evaluation

Three different kinds of bacteria such as *Escherichia coli* (*E.coli*, Gram negative), *Staphylococcus aureus* (*S.aureus*, Gram positive) and *Methicillin-resistant Staphylococcus aureus* (*MRSA*) were used to evaluate the antibacterial properties of FABA hydrogel. To be brief, 10 µL bacterial suspension containing 10^5^ bacteria was added to the surface of 300 µL hydrogel in a 48-well cell culture plate and incubated at 37 °C for 3 h. Subsequently, 100 µL of PBS was gently added into the cell culture plate to resuspend the bacteria. 5 µL of bacterial solution was coated on LB solid culture dish. After incubation at 37 °C for 12 h, the number of monoclone colonies in the culture dish was counted. The relative expression of bacteria is calculated by using image J software to count the area of bacteria in the plate, and then standardized by the control group.

Lipopolysaccharide (LPS, Sigma, L2880) induced RAW264.7 macrophages were used to detect the anti-inflammatory ability of FABA hydrogel. RAW264.7 macrophages were seeded in a 12-well cell culture plated. When the cell density reached 70%, changed the medium into DMEM supplemented with 10% FBS and 200 ng/mL LPS and continued to culture at 37 °C for 24 h. Then 100 µL of hydrogels were added to the cells and cultured for 24 h. Cells were washed with phosphate buffered saline (PBS) for once. Then total RNA was extracted with RNAiso Plus (cat No.9109, TaKaRa) and the cDNA was synthesized using the First-Strand cDNA Synthesis Kit (G486, abm) accordance with the manufacturer’s instructions. The gene expression of *IL-10* and *TNF-α* were quantified by qPCR (Novoprotein, Shanghai, China, E096) (Primers were shown in Additional file 1: Table S1).

### Normal and *MRSA*-infected wound repair evaluations in vivo

All mice were purchased from the Animal Centre in Xi’an Jiaotong University. They were housed under room temperature with standard light conditions (12/12 h dark and light cycles) and plenty of food and water. All mice experiments were approved by the Animal Care and Use Committee (ACUC) of Xi’an Jiaotong University (No.: 2021 − 1953).

Skin wounds with a diameter of 8 mm were made on the waist of 90 mice. Then the mice were randomly divided into five groups (3 M membrane, control, FA, FAB and FABA group, n = 18). Mice in 3 M (Tegaderm™ film, 3 M Health Care, USA) membrane, control, FA, FAB and FABA groups were respectively treated with 3 M membrane, normal saline, FA hydrogel, FAB hydrogel and FABA hydrogel on the wounds. Skin samples were collected at day 3, 7 and 14 respectively. The effect of FABA hydrogel on wound healing was evaluated according to wound healing time, wound area, H&E staining and immunofluorescence staining.

In order to further verify the antibacterial effect of hydrogel during skin repair, we constructed a skin repair model for wound bacterial infection. Drug resistant bacteria *MRSA* was used to evaluate the antibacterial activity of FABA hydrogel in the infected wound healing model. Similar to the method described above, skin wounds with a diameter of 8 mm were made on the waist of 48 mice. Then 10 µL of fresh *MRSA* bacterial (10^7^ CFU/mL) solution was added to the injured skin. Mice were randomly divided into four groups (n = 12). The mice in control, FA, FAB and FABA groups were separately treated with normal saline, FA hydrogel, FAB hydrogel and FABA hydrogel. On day 3 and day 7, skin samples were collected and vibrated in 1 mL PBS for 1 min to ensure that the *MRSA* at the wound site could be fully dissolved in PBS. Then 5 µL of bacterial solution was coated on Luria-Bertani (LB) solid culture dish. After inubation at 37 °C for 12 h, the number of monoclone colonies in the culture dish was counted. Wound skin tissues were used to evaluate the effect of FABA hydrogel on skin repair in *MRSA*-infected wound healing models by H&E and immunofluorescence staining.

### Statistical analysis

Statistical analysis was performed by using GraphPad Prism version 7 (GraphPad Software Inc.) for Windows. All statistical experimental results were expressed as mean ± SD. An unpaired two-tailed Student’s t test was used for comparison between two groups. And analysis of variance (ANOVA) was used to test for differences among groups. *P* values < 0.05 were considered statistically significant.

## Supplementary information

**Additional file 1**: **Fig S1.** The SEM mapping of BGNCu, BGNCu@AL, FAB hydrogel and FABA hydrogel. **Fig S2. **The FTIR spectra of FA, FAB and FABA hydrogels. The results showed that the disappearance of the peak at 1735 cm^-1^ in FABA hydrogel indicated the successful reaction between -CHO in F127-CHO and -NH_2_ in AL. **Fig S3. **G' and G'' of FAB and FABA hydrogel when the step strain switched from 1% to 1000% at 35°C.The G' and G'' of FAB and FABA hydrogel at 35°C. **Fig S4. **Degradation rate of FABA hydrogel in vitro. **Fig S5.** Degradation rate of FABA hydrogels in vivo. **Fig S6.** Histological evaluation of the effect of FABA hydrogel on MRSA infection-induced wound healing. After 5 μL of fresh MRSA bacterial solution was added to the injured skin, skin samples in control, FA, FAB and FABA groups were collected for HE staining on day 3 and day 7. The normal skin tissue was used as a control. **Fig S7.** Evaluation of tissue toxicity of FABA hydrogel in vivo. Main tissue organs were collected for H&E staining to evaluate the biosafety of the hydrogel after 3 d or 7 d treatment with FA, FAB and FABA hydrogel in the wound healing model mice. The normal tissues were used as control. **Fig S8.** Comparison of the anti-MRSA performance of FABA hydrogel with commercial antibiotics methicillin and vancomycin. Imaging of wound healing on day 0, 3 and 7 after methicillin, vancomycin and FABA treatment. Schematic diagram showed the wound area size on day 0, 3 and 7. The wound area on day 0, 3 and 7. Statistics of skin wound healing rate on day 3 and 7. The antibacterial activity of methicillin, vancomycin and FABA hydrogels on MRSA in the infection-induced wound healing at day 3 and 7. The relative clone numbers of MRSA on day 3 and 7. *P <0.5, **P<0.01, ***P<0.001 and n.s means no significance. **Table S1**. Information of qPCR primers.
