# Supplementary material for: Multifunctional antibacterial bioactive nanoglass hydrogel for normal and MRSA infected wound repair
Source: J Nanobiotechnology. 2023 May 21;21:162. doi: 10.1186/s12951-023-01929-9 (PMC10200057; doi:10.1186/s12951-023-01929-9)
Supplement: Supplementary file 1 — Additional file 1: Fig S1. The SEM mapping of BGNCu, BGNCu@AL, FAB hydrogel and FABA hydrogel. Fig S2. The FTIR spectra of FA, FAB and FABA hydrogels. The results showed that the disappearance of the peak at 1735 cm-1 in FABA hydrogel indicated the successful reaction between -CHO in F127-CHO and -NH2 in AL. Fig S3. G' and G'' of FAB and FABA hydrogel when the step strain switched from 1% to 1000% at 35°C.The G' and G'' of FAB and FABA hydrogel at 35°C. Fig S4. Degradation rate of FABA hydrogel in vitro. Fig S5. Degradation rate of FABA hydrogels in vivo. Fig S6. Histological evaluation of the effect of FABA hydrogel on MRSA infection-induced wound healing. After 5 μL of fresh MRSA bacterial solution was added to the injured skin, skin samples in control, FA, FAB and FABA groups were collected for HE staining on day 3 and day 7. The normal skin tissue was used as a control. Fig S7. Evaluation of tissue toxicity of FABA hydrogel in vivo. Main tissue organs were collected for H&E staining to evaluate the biosafety of the hydrogel after 3 d or 7 d treatment with FA, FAB and FABA hydrogel in the wound healing model mice. The normal tissues were used as control. Fig S8. Comparison of the anti-MRSA performance of FABA hydrogel with commercial antibiotics methicillin and vancomycin. Imaging of wound healing on day 0, 3 and 7 after methicillin, vancomycin and FABA treatment. Schematic diagram showed the wound area size on day 0, 3 and 7. The wound area on day 0, 3 and 7. Statistics of skin wound healing rate on day 3 and 7. The antibacterial activity of methicillin, vancomycin and FABA hydrogels on MRSA in the infection-induced wound healing at day 3 and 7. The relative clone numbers of MRSA on day 3 and 7. *P <0.5, **P<0.01, ***P<0.001 and n.s means no significance. Table S1. Information of qPCR primers. [file 12951_2023_1929_MOESM1_ESM.doc]

**Multifunctional Antibacterial Bioactive Nanoglass Hydrogel for Normal and *MRSA* Infected Wound Repair**

Long Zhang ^a,b^*, Wen Niu ^b^, Yuyao Lin ^c^, Junping Ma ^b^, Tongtong Leng ^b^, Wei Cheng ^b^, Yidan Wang ^b^, Min Wang ^d^, Jingya Ning ^a^, Shuanying Yang ^a*^ , Bo Lei ^b^

*^a^ Department of Respiratory and Critical Care Medicine, The Second Affiliated Hospital of Xi’an Jiaotong University, Xi’an, 710004, China*

*^b^ Frontier Institute of Science and Technology, Xi’an Jiaotong University, Xi’an 710054, China*

^c^ Department of Plastic, Aesthetic and Maxillofacial Surgery, The First Affiliated Hospital of Xi'an Jiaotong University, Xi'an 710061, China

*^d^ Honghui Hospital, Xi'an Jiaotong University, Xi’an 710068, China*

* To whom correspondence should be directed

E-mail: longzhang@xjtu.edu.cn (Long Zhang)

E-mail: yangshuanying@xjtu.edu.cn (Shuanying Yang)

**Synthesis of F127-TsCl and F127-CHO**

F127-TsCl and F127-CHO were synthesized according to our previous description. Briefly, 6.3 g of F127 (MW=12600) was dissolved in 35 mL dichloromethane, and then 0.695 mL triethylamine (5 mmol) was added. Subsequently, 10 mL of anhydrous dichloromethane that dissolved 0.9535 g of p-toluenesulfonyl chloride was slowly added into the above solution under the nitrogen atmosphere. After 2 days reaction at room temperature, a certain amount of hydrochloric acid and saturated sodium bicarbonate were added to extract the impurities. Finally, the sample was precipitated with ether and left to stand overnight before suction filtration. After vacuum drying, F127-TsCl intermediate was obtained. Dissolve 2.5 g of F127-TsCl, 0.109 g of 4-hydroxybenzaldehyde and 0.12 g of potassium carbonate in DMF. After reacting at 80 °C for 3 days , appropriate amounts of dichloromethane and magnesium sulfate were added to extract and remove the organic phase and water. Precipitated the sample with ether, and dry the sample in vacuum to obtain F127-CHO.

**Synthesis of BGNCu and BGNCu@AL**

2 g of dodecylamine (DDA) was dissolved in 40 ml ethanol and 12.5 ml water. After complete dissolution, it was placed in a 45°C water bath and stirred for 30 minutes. Then 1 mL of ethyl orthosilicate (TEOS) was added dropwise and stirred for 30 min; 76 μL of triethyl phosphate (TEP) was added and continue stirring for 30 min. Added 0.55 g calcium nitrate and stirred for 15 minutes. Then 0.10 g of copper nitrate was added and stirred for 3 h. Centrifuge to obtain blue precipitate and wash 4 times with ethanol and deionized water respectively. BGN-Cu nanoparticles were obtained by freeze-drying and high temperature calcination. Then 10 mg/mL of alendronate (AL) was dissolved in 0.05 M, pH = 4 sodium acetate buffer. After a certain amount of BGNCu (5mg/mL) was added, stirred for 24 h and centrifuged to obtain the precipitate. Then wash the precipitate with deionized water for 3 times. BGNCu@AL powder was obtained by freeze-drying.

**Preparation of F127-CHO-BGNCu@AL (FABA) gel**

For FABA hydrogel preparation, BGNCu@AL was dispersed in PBS at the concentration of 1mg/mL. The ultrasonic treatment of the sample makes BGNCu@AL completely dispersed. Then, F127-CHO powder and BGNCu@AL dispersion were mixed according to a mass-volume ratio of 35%. After mixing thoroughly, place the sample in a 55°C water bath for 5 h to obtain F127-CHO-BGNCu@AL (FABA) gel.

**Biocompatibility and safety of FABA hydrogel *in vitro***

Red blood cells from Kunming mice were used to detect the hemolytic property of FABA hydrogel. Fresh mouse blood was taken into 1.5 mL EP tube by tail cutting method, centrifuged with 0.5% heparin solution at 1000 rpm for 10min, and washed with PBS for 5 times to obtain red blood cells. Then 200 μL hydrogel was added in a 48-well cell culture plate and placed the plate at 37°C for 5 min. After the hydrogel was solidified, 500 μL diluted red blood cell suspension was added into the culture plate. Equal volume diluted blood cells suspension and cells suspension containing 0.1% TritonX-100 were used as control groups. After incubation at 37°C for 1 h, the cell suspension in the culture dish was transferred to a 1.5 mL EP tube and centrifuged at 1000 rpm for 10 min. Photos were taken to evaluate the hemolysis of red blood cells. Then transferred 100 μL of the supernatant to a 96-well plate to detect the absorbance of the liquid at 600 nm. (Hemolysis rate (%) =[(ODsample-ODPBS)/(ODtriton-ODPBS)] × 100%). After that, the cells were re-suspended, and the morphological characteristics of cells were observed under the microscope. L929 cells were used to detect the cytotoxicity of the Hydrogels. In the 4°C environment, different volumes (2 μL, 5 μL, 10 μL, 20 μL) of hydrogels were added to the 96-well cell culture dish (100 μL cell culture medium). Then put the culture dish in a 37°C incubator to solidify the hydrogels. 100 μL of L929 cell suspension with 7000 cells was added to the culture dish containing the solidified hydrogels. After incubation at 37°C for 24 h, cell viability was measured using CCK8 assay Kits (C0005, TargetMol, USA) according to the manufacturer’s instructions.

**Amount of NH_2_ detect**

10 mg of BGNCu@AL was dissolved in 1 mL of glacial acetic acid, and then 13 mL of deionized water and 2 mL of hydrochloric acid solution (6 mol/L) were added. After the complete dissolution of BGNCu@AL, 0.067 g of potassium bromide was added. Subsequently, stir the solution in an ice bath environment, cool the liquid to 0-5 ℃, and add 0.1mol/L of standard solution of sodium nitrite dropwise until the starch potassium iodide test paper turns blue and does not fade for five minutes. Record the volume of the solution used.

Amino value%= (C*M*V*1/1000) / G

C = The concentration of standard solution of sodium nitrite (mol/L)

V = Volume of sodium nitrite standard solution consumed (mL)

G = Mass of BGNCu@AL weighed (g)

M = Molecular mass of amino group

Amino value% = (0.1*16*0.04*1/1000) / (10*1/1000) * 100% = 0.64%


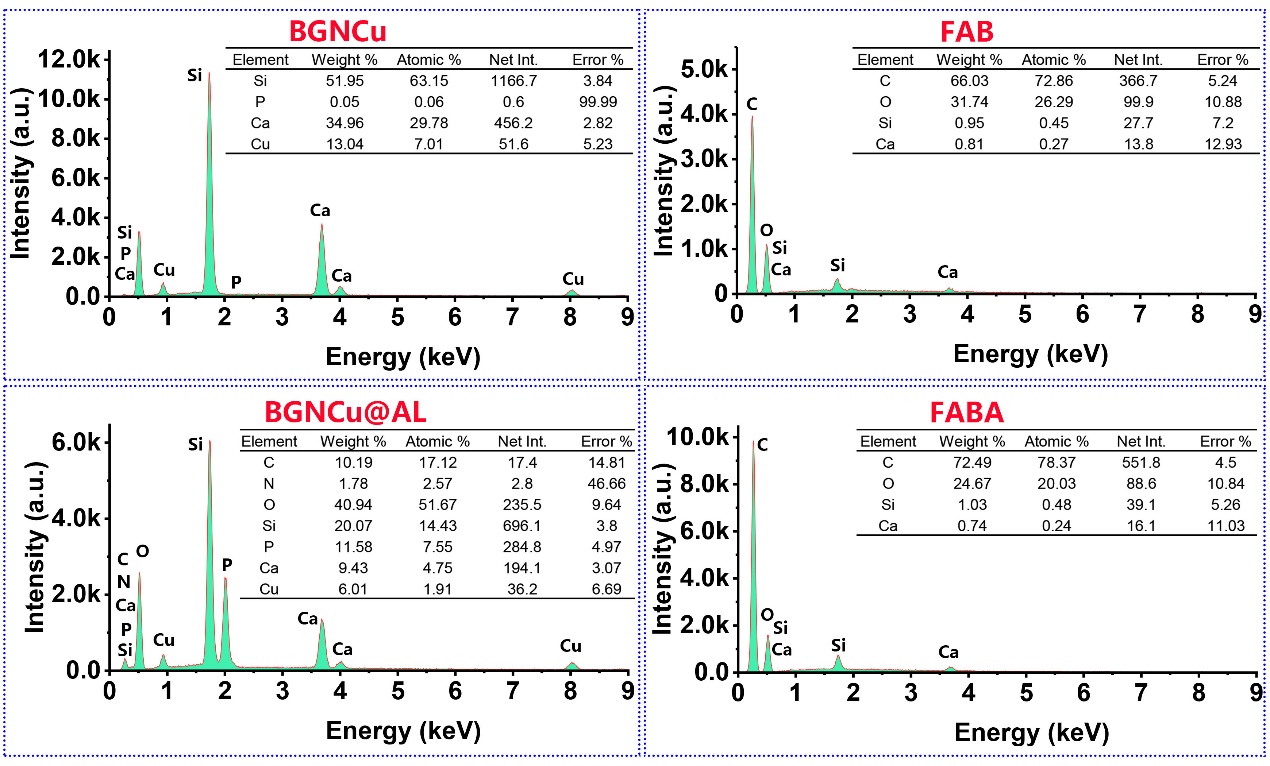


Fig S1. The SEM mapping of BGNCu, BGNCu@AL, FAB hydrogel and FABA hydrogel.


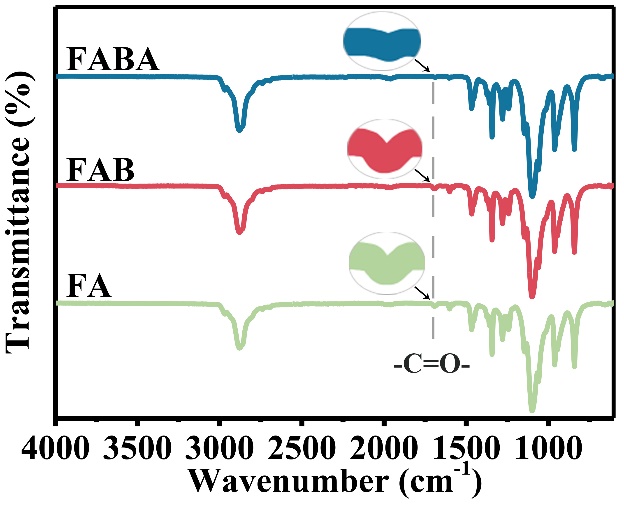


Fig S2. The FTIR spectra of FA, FAB and FABA hydrogels. The results showed that the disappearance of the peak at 1735 cm^-1^ in FABA hydrogel indicated the successful reaction between -CHO in F127-CHO and -NH_2_ in AL.


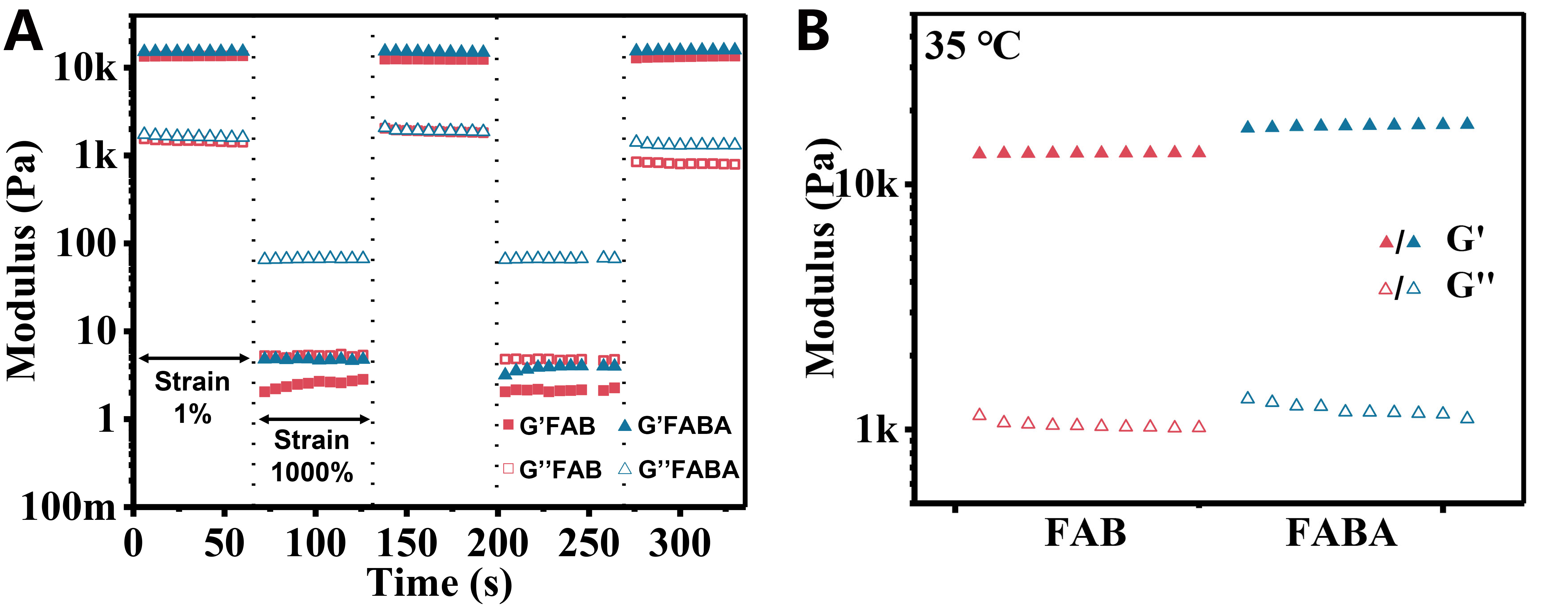
Fig S3. (A) G' and G'' of FAB and FABA hydrogel when the step strain switched from 1% to 1000% at 35°C. (B) The G' and G'' of FAB and FABA hydrogel at 35°C.


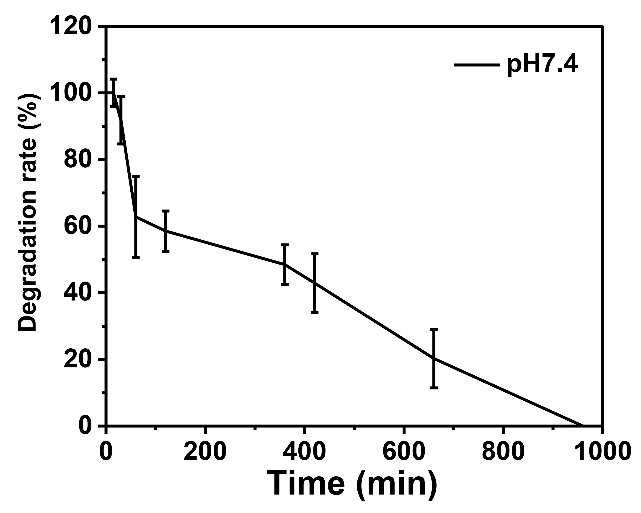


Fig S4. Degradation rate of FABA hydrogel *in vitro*.


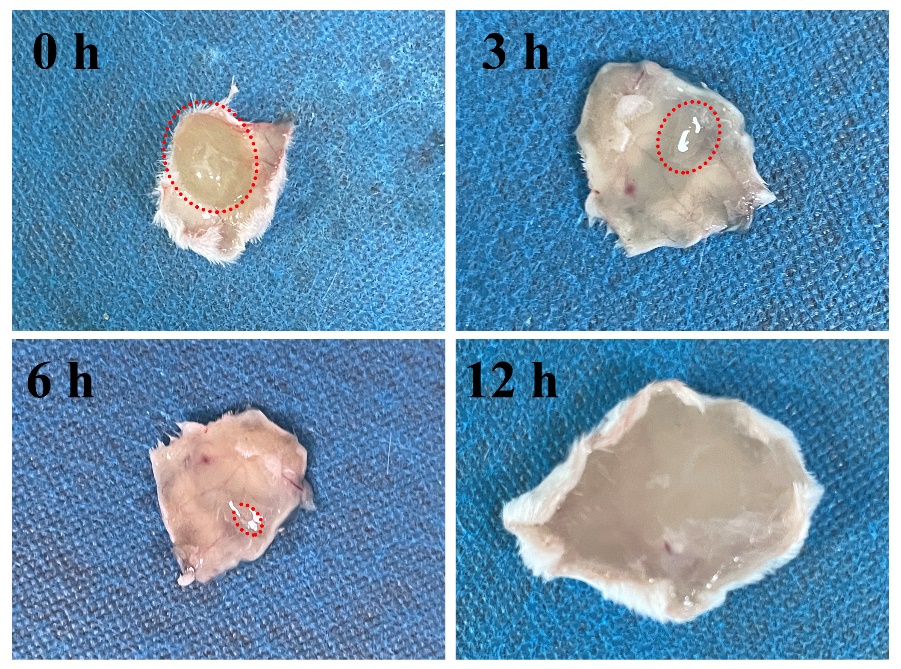


Fig S5. Degradation rate of FABA hydrogels *in vivo*.


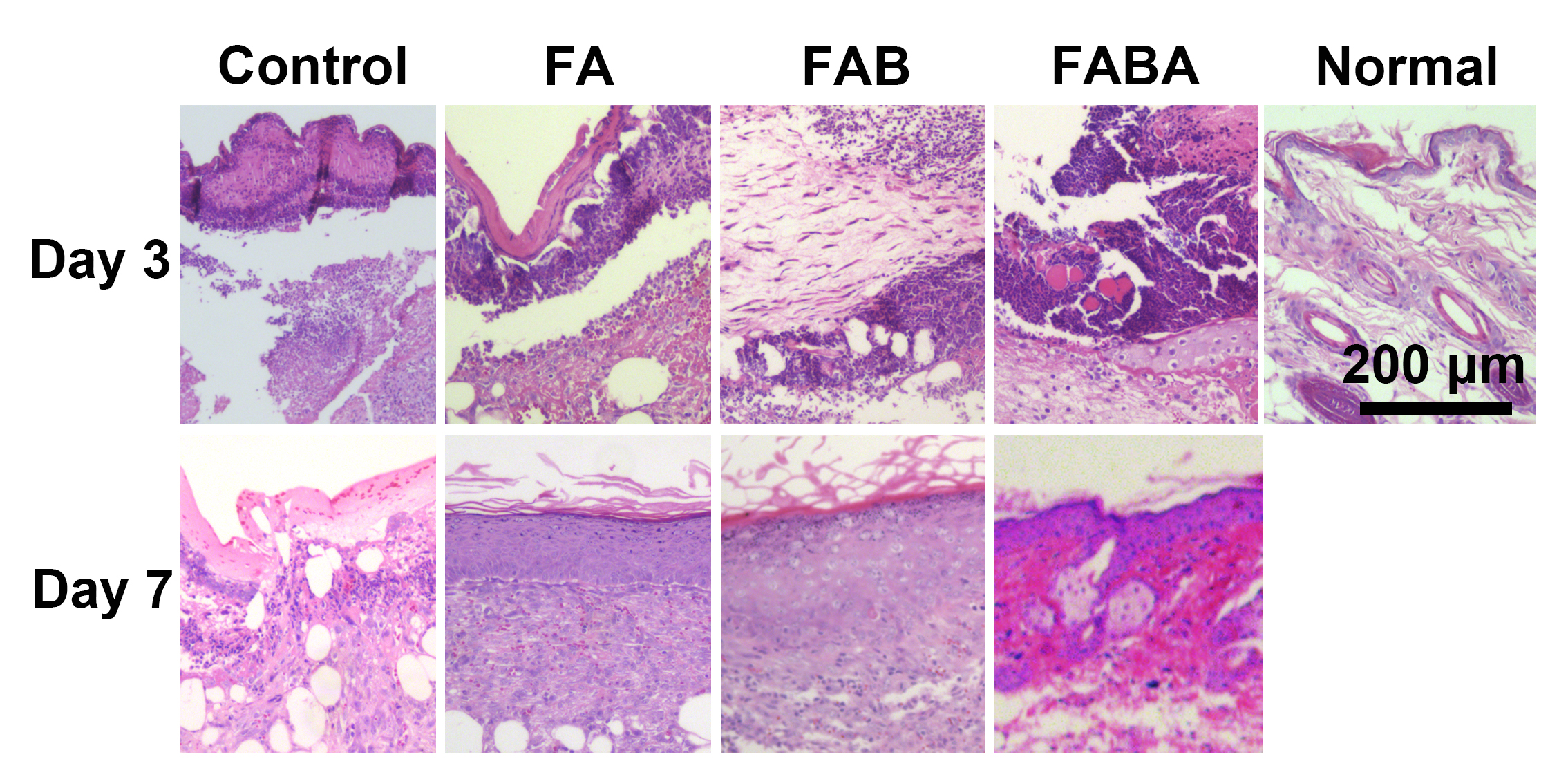


Fig S6. Histological evaluation of the effect of FABA hydrogel on MRSA infection-induced wound healing. After 5 μL of fresh MRSA bacterial (106 CFU/mL) solution was added to the injured skin, skin samples in control, FA, FAB and FABA groups were collected for HE staining on day 3 and day 7. The normal skin tissue was used as a control.


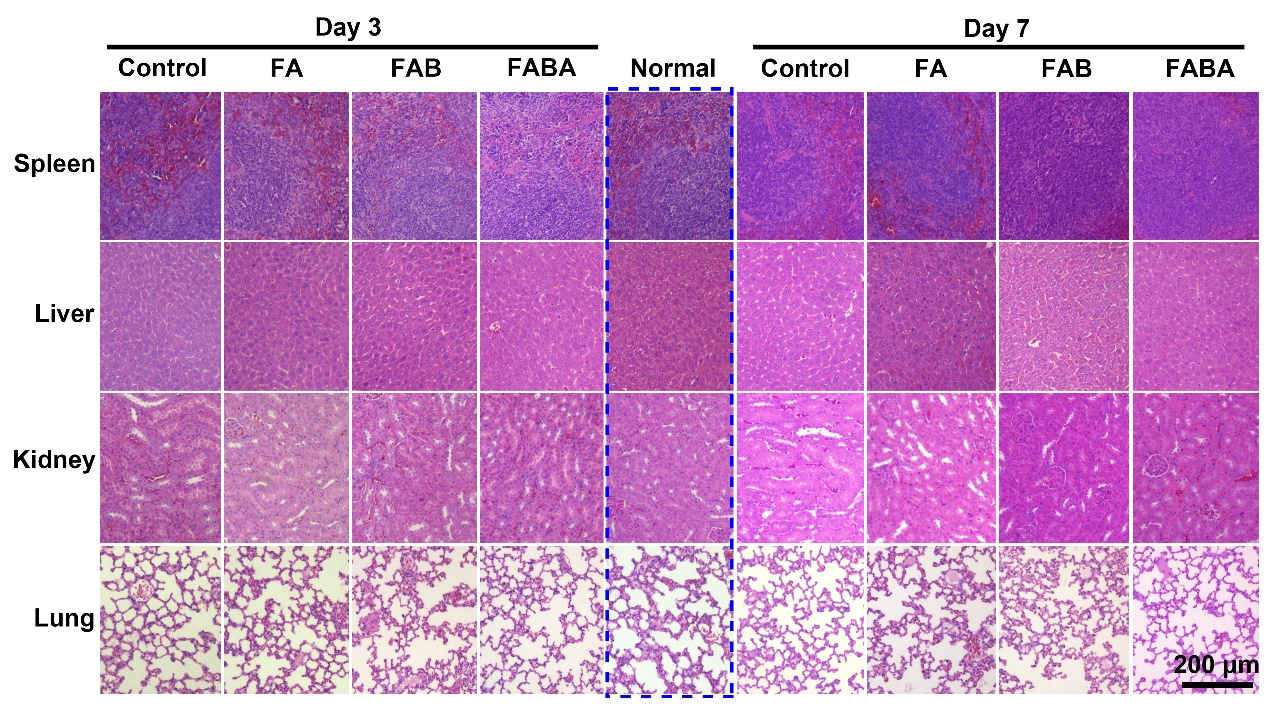


Fig S7. Evaluation of tissue toxicity of FABA hydrogel *in vivo*. Main tissue organs (spleen, liver, kidney and lung) were collected for H&E staining to evaluate the biosafety of the hydrogel after 3 d or 7 d treatment with FA, FAB and FABA hydrogel in the wound healing model mice. The normal tissues were used as control (n=6).


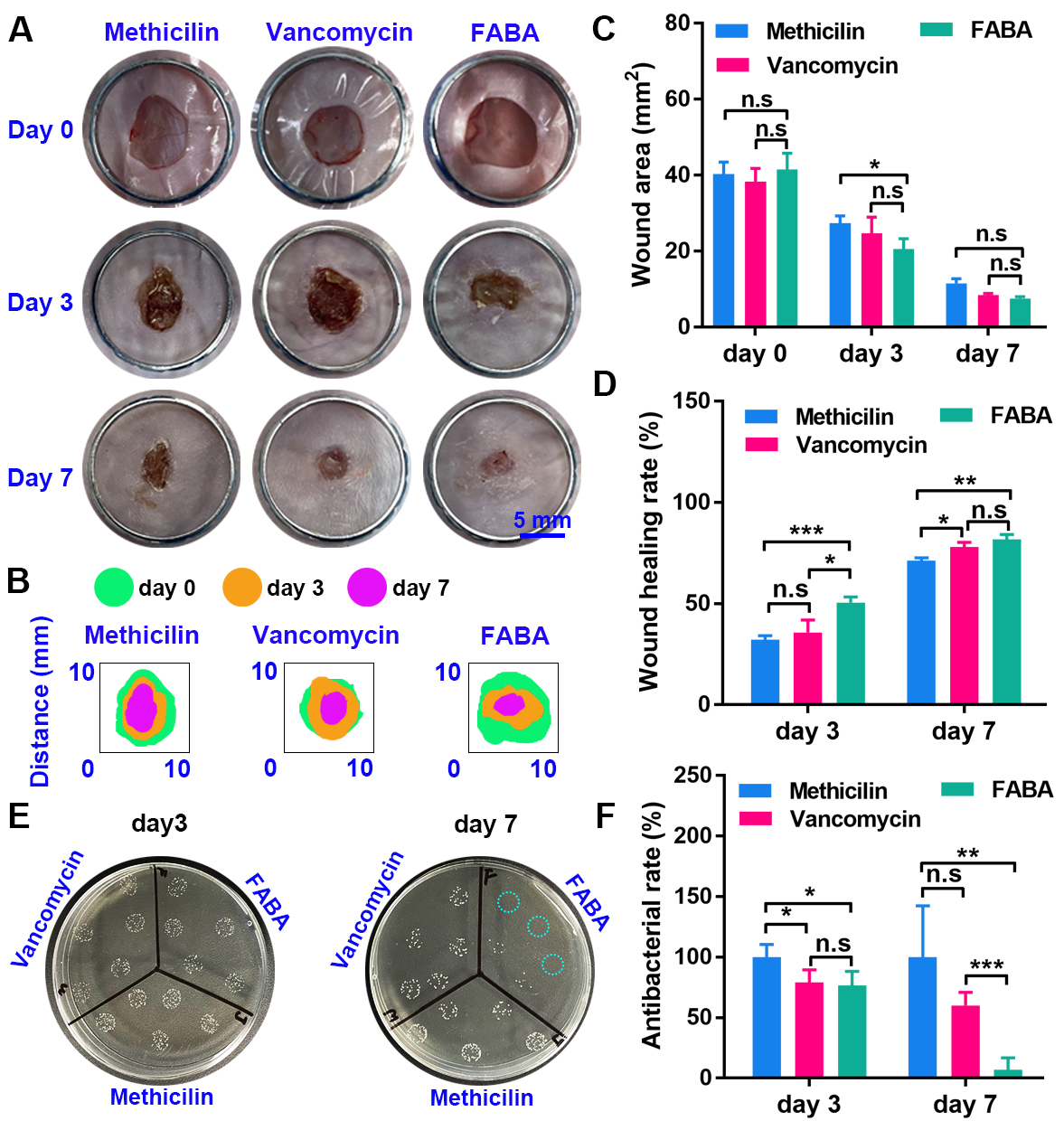


Fig S8. Comparison of the anti-MRSA performance of FABA hydrogel with commercial antibiotics methicillin and vancomycin. (A) Imaging of wound healing on day 0, 3 and 7 after methicillin, vancomycin and FABA treatment. (B) Schematic diagram showed the wound area size on day 0, 3 and 7. (C) The wound area on day 0, 3 and 7. (D) Statistics of skin wound healing rate on day 3 and 7. (E) The antibacterial activity of methicillin, vancomycin and FABA hydrogels on MRSA in the infection-induced wound healing at day 3 and 7. (F) The relative clone numbers of MRSA on day 3 and 7 (n=3). **P <0.5, **P <0.01, ***P<0.001* and n.s means no significance.

Table S1. Information of qPCR primers.

| Name |  | Sequence (5' to 3') |
| --- | --- | --- |
| 18S forward primer |  | CGGCTACCACATCCAAGGAA |
| 18S reverse primer |  | GCTGGAATTACCGCGGCT |
| IL-10 forward primer |  | GAGAGCTGCAGGGCCCTTTGC |
| IL-10 reverse primer |  | CTCCCTGGTTTCTCTTCCCAAGACC |
| TNF-α forward primer |  | ACCCTCACACTCAGATCATCTTC |
| TNF-α reverse primer |  | TGGTGGTTTGCTACGACGT |
